# Natural Active Ingredients for Poly (Lactic Acid)-Based Materials: State of the Art and Perspectives

**DOI:** 10.3390/antiox11102074

**Published:** 2022-10-20

**Authors:** Andrea Lombardi, Andrea Fochetti, Pamela Vignolini, Margherita Campo, Alessandra Durazzo, Massimo Lucarini, Debora Puglia, Francesca Luzi, Marco Papalini, Monia Renzi, Andrea Cavallo, Roberta Bernini

**Affiliations:** 1 Department of Agriculture and Forest Sciences (DAFNE), University of Tuscia, Via San Camillo de Lellis, 01100 Viterbo, Italy; 2 Phytolab, Department of Statistics, Informatics, Applications “G. Parenti”, DiSIA, University of Florence, Via Ugo Schiff 6, 50019 Sesto Fiorentino, Italy; 3 CREA-Research Centre for Food and Nutrition, Via Ardeatina 546, 00178 Rome, Italy; 4 Civil and Environmental Engineering Department, University of Perugia, Strada di Pentima 4, 05100 Terni, Italy; 5 Department of Materials, Environmental Sciences and Urban Planning (SIMAU), Polytechnic University of Marche, Via Brecce Bianche 12, 60131 Ancona, Italy; 6 BIORICERCHE S.R.L. Loc. Ferro di Cavallo, 58034 Castell’Azzara, Italy; 7 Dipartimento di Scienze della Vita, Università di Trieste, Via L. Giorgieri 10, 34127 Trieste, Italy; 8 CERTEMA S.c.a.r.l., 58044 Cinigiano, Italy; 9 Bioscience Research Center Srl., Via Aurelia Vecchia 32, 58015 Orbetello, Italy

**Keywords:** active ingredients, phenols, terpenes, essential oils, antioxidant activity, antimicrobial activity, poly (lactic acid) (PLA), PLA-based materials, circular economy, green chemistry, sustainable materials

## Abstract

This review describes the state of the art in the field of poly (lactic acid) (PLA)-based materials activated by natural compounds and extracts (active ingredients, AIs) from plant sources for food and biomedical applications. With a multidisciplinary approach, after a description of the synthesis and properties of PLA, special attention was paid to the chemical properties and unconventional extraction technologies of AIs used for PLA activation. Innovative techniques for the incorporation of AIs into PLA; characterization and the antioxidant and antimicrobial properties of the novel materials were discussed. In view of future perspectives, this study has evidenced that some aspects need to be further investigated from joint research between academia and industry, according to the green chemistry principles and circular economy strategy.

## 1. Introduction

Reactive oxygen species (ROS) and reactive nitrogen species (RNS) such as hydrogen peroxide (H_2_O_2_), hypochlorous acid (HClO), superoxide radical (O_2_^•−^), hydroxyl radical (HO^•^), nitric oxide (NO^•^), and peroxynitrite (ONOO^−^) are the main agents responsible for oxidative processes.

In the human body, free radicals are mainly produced by both metabolic processes and external factors, such as environmental pollution, cigarette smoke, and UV exposure. If not slowed down by antioxidants, they are responsible for the onset of oxidative stress, a status involved in the development of chronic and oxidative stress-related disorders, including immune deficiency, cardiovascular and neurodegenerative diseases [1,2,3], type 2 diabetes, cancer [4,5,6], and cellular aging [7].

In foods, free radicals affect color, texture, taste, and shelf-life, causing a relevant loss of nutritional value and safety [8] and leading to a rapid decay of their organoleptic properties, with consequent unpleasant sensory characteristics [9,10].

Another cause of food deterioration is microbial contamination [11]. The incorrect management of food at any point in the production chain can represent a potential danger for consumers. Despite some management systems, such as Hazard Analysis and Critical Control Points (HACCP), that are consolidated for food safety, the data relating to diseases caused by the consumption of spoiled foods are of concern. In 2015, the World Health Organization (WHO) reported that 420,000 deaths/year were due to food contamination and that at least one in ten people had health problems after consuming contaminated food [12]. 

In addition to food safety, food availability is another important aspect. A recent study revealed that about 14% of food is lost between the harvest and commercialization phases [13]. This has led the United Nations to consider food waste as a problem to be solved in the immediate future. In fact, in the 2030 Agenda for Sustainable Development, food security and availability are addressed in the 17 Sustainable Development Goals (SDGs) [UN, 2015] and, among them, SDG 12 promotes the efficient management and use of natural resources as well as the reduction of waste production by halving the global per capita food waste. For this reason, the Food and Agriculture Organization (FAO) and United Nations Environment Programme (UNEP) have introduced two indices to monitor food waste: the Food Loss Index (FLI), which focuses on the food supply chain, and the Food Waste Index (FWI), which focuses on “demand-oriented” aspects such as stages of retail and final consumption [14]. 

In addition to limiting waste, another global priority is to ensure safe food for all populations, as evidenced by SDG 2, “zero hunger”. One of the most valuable strategies to both assure food safety levels and reduce waste is to increase the shelf-life of food products. 

In this context, active food packaging represents an interesting research area both in the academic and industrial fields [15]. It consists of incorporating an active ingredient (AI) inside the polymer matrix of the packaging material. AI can be a pure compound, or a mixture of compounds found in an extract obtained from plant materials. The controlled release of the AI from the polymer matrix to the food surface prolongs its shelf-life increasing safety and preserving the quality. Active materials and objects are “materials and objects intended to extend the shelf-life or maintain or improve the conditions of packaged food products” [16]. Food additives are reported in a list and generally exhibit antioxidant and antimicrobial activity, thus protecting the packaged food and hindering its degradation. The most commonly used AIs are benzoic acid, butylated hydroxyanisole (BHA), and 2,6-di-*tert*-butyl-4-methylphenol (BHT).

A valid and safe alternative to the use of synthetic antioxidant and antimicrobial agents is offered by AI recovered from plant materials and waste biomasses in combination with polymers such as polypropylene, poly (vinyl alcohol), poly (vinyl alcohol-co-ethylene), and poly (lactic acid). Thymol [17], gallic acid, quercetin [18,19], hydroxytyrosol [20,21], Olea extracts [22], red grape seeds extracts, tomato extracts [23], and almond skin extracts [24] are some examples of AIs which confer to the materials antioxidant and antimicrobial activity.

Among polymeric materials used for both food and biomedical applications, poly (lactic acid) (PLA) has become the most widely used due to its versatility, biocompatibility, and compostability properties [25,26]. A recent economic analysis has estimated that the global market will grow from 1.1 billion dollars in 2020 to 1.756 billion dollars in 2025 [27]. 

Currently, innovation focuses on transposing active packaging technology into biodegradable materials to reduce plastic waste, which is a major cause of environmental pollution. In particular, the use of innovative PLA-based materials incorporating AI represents an alternative to mitigate the accumulation of plastics and the production of food waste [28,29,30,31,32,33].

Based on the literature data collected, with an integrated and multidisciplinary approach, this review aims at describing the state of the art of sustainable PLA-based materials, including AIs used for applications ranging from food to the biomedical field. Specifically, after a brief overview of traditional and innovative strategies for PLA synthesis, a wide variety of AIs found in nature are described, turning particular attention to phenols and terpenes used and usable for PLA activation. After the description of the innovative methods for extracting AIs, advanced techniques to prepare activated PLA-based materials are reported. Finally, antioxidant and antimicrobial properties, as well as environmental aspects, are discussed in view of future perspectives of academic and industrial research.

## 2. PLA Synthesis and Structure

**Figure 1:**
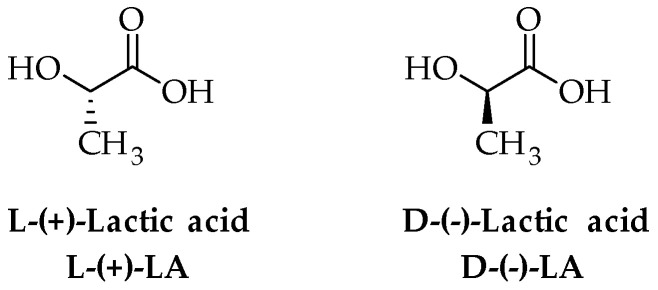
Structure of enantiomers of LA.

Poly (lactic acid) (PLA) is a highly versatile thermoplastic polymer that belongs to the family of polyesters obtained by α-hydroxyacids. PLA shows high biodegradability and biocompatibility [34], along with good mechanical properties that make it an ideal candidate for biomedical and packaging uses. Structurally, PLA is a hydrophobic chiral polymer of lactic acid (2-hydroxypropionic acid, LA), which is a naturally occurring α-hydroxyacid with two optical forms, namely L-(+)-LA and D-(−)-LA (Figure 1).

Monomers of LA are produced mainly in two ways: bacterial fermentation and chemical synthesis [35]. Fermentation by lactic acid bacteria is usually preferred because of the possibility to obtain chemically and optically pure LA, while high manufacturing costs and limited production capacity pose severe limitations to the employment of the chemical pathway [36]. Moreover, since LA can be derived from the fermentation of sugar in corn and sugarcane [37]], PLA can be 100% obtained from renewable resources [38].

**Scheme 1:**
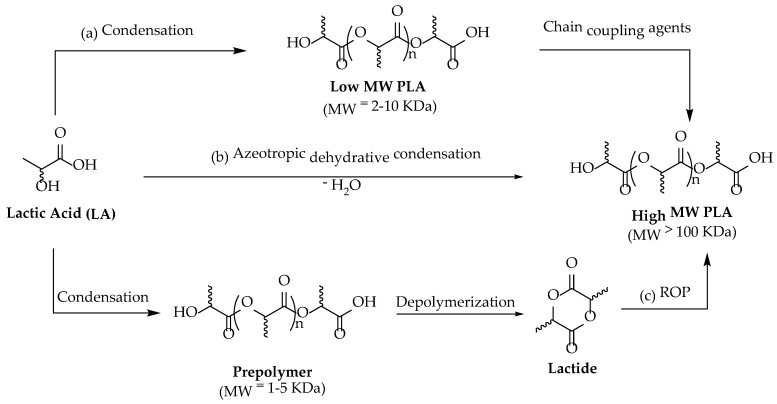
Possible routes of PLA production.

LA can be polymerized into three stereoisomers of PLA, usually referred to as poly (L-Lactide) (PLLA), poly (D-Lactide) (PDLA), and poly (DL-Lactide) (PDLLA). The crystal structure and properties of PLA depend on its molecular weight and composition. Homopolymers such as PLLA and PDLA are semicrystalline with a melting point of ca. 180 °C and a glass transition temperature of ca. 55 °C, whereas PDLLA has an amorphous structure [39]. LA can be used to synthesize PLA with variable molecular weight, but only high-molecular-weight PLA (about 100,000 Da) has a commercially relevant value due to its potential employment in the packaging industry [40]. PLA can be produced mainly via three different routes using LA as starting material: (a) direct condensation polymerization; (b) azeotropic dehydrative condensation; and (c) ring-opening polymerization (ROP) [41] (Scheme 1). 

In the direct condensation method, LA is heated at 50 °C under vacuum, and water is constantly removed from the reaction medium to avoid hydrolysis of the obtained polymer. Dehydration becomes more difficult during the final stage of polymerization because of the increased viscosity of the polymeric melt. This process has two main disadvantages: first, only low-molecular-weight PLA (<100 kDa) with low mechanical properties is obtained; second, it requires extended reaction time [42]. For such reasons, direct poly-condensation for the synthesis of PLA is poorly used [43].

Azeotropic dehydrative polycondensation allows for an increase in the molecular weight of PLA. This method, first employed by Mitsui Chemicals Co. (Tokyo, Japan), does not require catalysts or chain extenders [25] that are necessary to increase the molecular weight of the oligomers provided by polycondensation. However, long reaction times and the use of large amounts of high-boiling-point solvents make this procedure unattractive from an ecological point of view [44].

**Figure 2:**
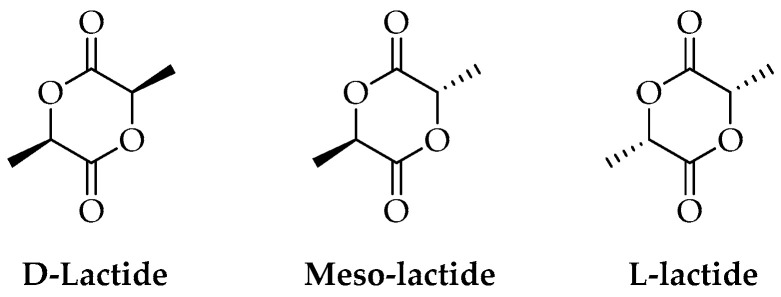
Stereoisomers of lactide.

Ring-opening polymerization is by far the most common route to obtain high-molecular-weight polymers [40,45]. This method consists of converting lactide (the cyclic dimer of lactic acid) to PLA (Figure 2).

**Figure 3:**
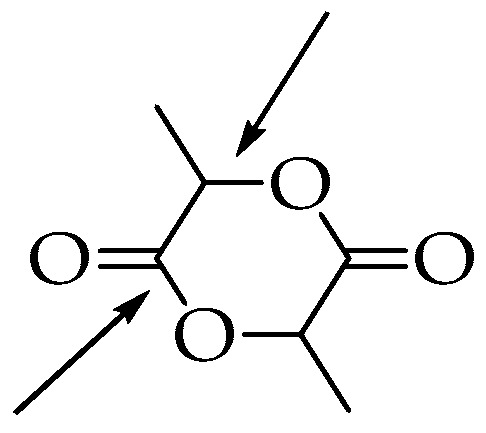
Different opening positions of the lactide ring.

Lactide can be obtained and purified in several different ways, all of which require high temperatures and are often performed under reduced pressure. In 2007, Wang and co-workers reported an 86% yield of PLA by heating LA to 180–206 °C at 95 kPa for 7 h in the presence of zinc and tin catalysts [46]. In the process, lactic acid was first dehydrated and polycondensed, then lactide was obtained by the catalytic depolymerization of short PLA chains under reduced pressure, and eventually, the purified lactide was polymerized at high temperatures. Based on the different positions where the lactide ring is opened (Figure 3), three different mechanisms can be described: cationic, anionic, and coordinative.

**Scheme 2:**
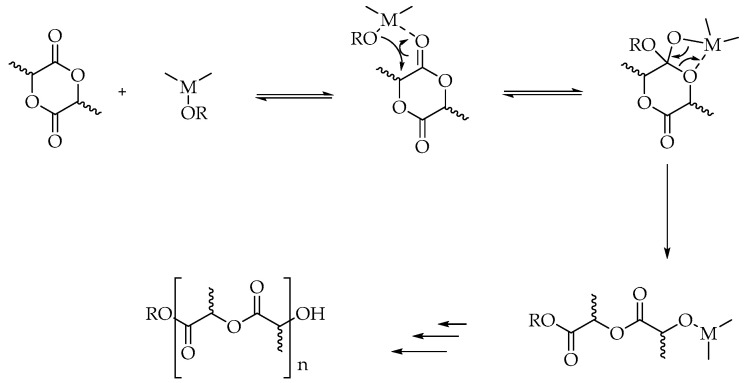
Coordination-insertion mechanism of ROP of lactide.

In the cationic ring opening, methyl triflate is used as the polymerization initiator; however, the mechanism involves substitution reactions on chiral centers so the products can undergo racemization. This effect, along with the fact that only low-molecular-weight PLA is obtained, significantly limits the use of this method on an industrial scale [39]. In the anionic mechanism, where alkali metal alkoxides represent the initiators, less than 5% racemization is observed if the starting lactide is enantiopure [47,48]. The process is characterized by high efficiency, good reaction rate, and a small number of side products [26]. In the coordinative mechanism (Scheme 2), there is an initial combination between the lactide after the acyl-oxygen cleavage and the initiator. The most commonly used initiators on the industrial scale are zinc, aluminum, titanium, tin, and stannous compounds [39,45].

Recently, great progress has been made in the field of bioproduction of PLA following the fermentation process. Microorganisms can be metabolically engineered to enhance the production of PLA and its copolymers. For example, *Escherichia coli* with propionate-Coenzyme A transferase and PHA synthase has been successfully used to obtain 43% wt. of PLA using glucose as the substrate [49]. Nonetheless, this strategy shows an important issue: as the polymer content increases, its molecular weight decreases, limiting the process to the synthesis of relatively short chains of PLA.

Another emerging valuable alternative is environmentally benign enzymatic polymerization, which can be carried out under mild conditions. In 2009, Lassalle and Ferreira reported a 55% yield from the conversion of LA into PLA using immobilized *Candida antarctica* Lipase B (CAL-B). However, it should be underlined that only extremely low-molecular-weight polymer was recovered from the reaction medium (about 400 Da). A slight improvement in the reaction yield was attained by Chanfreau et al., who used L-lactide in the presence of Novozyme-435 to achieve a 63% conversion into high-molecular-weight PLA (>30 kDa) at 90 °C using an ionic liquid as a solvent [50].

## 3. Active Ingredients

### 3.1. Diffusion in Nature

In recent years, plant extracts have attracted great interest for the potential large-scale application of AIs. A wide range of biologically active compounds endowed with several functions and properties [51,52] can be synthesized at various concentrations in all plant organs or parts—i.e., leaves, flowers, fruits, roots, barks, tubers, rhizomes—and there is an ever-growing effort to take advantage of the nutraceutical features of natural products [53,54,55].

In general, an AI is defined as a naturally occurring compound able to interact, throughout the food chain, with one or more components of living tissue, showing an effect on human health [56]. The variety of AIs is reflected in the endless arrangements that can be established between the main constituting functional groups. The combined and concerted actions of nutritional components and biologically active compounds are marked as an indicator of a possible beneficial role for health. 

The use and applications of AIs cover a wide range of sectors, in particular those regarding nutraceuticals [57,58]. Plants are a well-established rich source of antioxidants and antimicrobial molecules [59], and notable examples of plant families containing high antioxidant bioactive principles include fruits, such as blackberries, strawberries, blueberries, black currants, walnuts, pomegranates, and others (*Rosaceae* (i.e., dog rose, strawberry, raspberry, blackberry, sour cherry), *Empetraceae* (crowberry), *Ericaceae* (blueberry), *Grossulariaceae* (black currant), *Juglandaceae* (walnut), *Asteraceae* (sunflower seed), *Punicaceae* (pomegranate), and *Zingiberaceae* (ginger)) [60].

Only a small fraction of the known plant species on earth (estimated at 250,000–500,000) have been studied for the presence of antioxidants and antimicrobial molecules, and only 1–10% are utilized by humans [61]. Most of the identified substances with antioxidant and/or antimicrobial characteristics belong to the plant kingdom, for instance, vitamins A, C, E, and phenolic compounds such as flavonoids, tannins, and lignans. These compounds are mainly synthesized to meet physiological needs such as defense against toxic agents, parasites, inadequate environmental conditions, ultraviolet rays, attraction of pollinators, structural support, and regulation by phytohormones. A particularly important function of secondary metabolism is the protection from oxidative stress; in fact, plants that have a marked secondary metabolism tend to be richer in these substances. Natural antioxidants, widely present in foods such as fruits and vegetables, mainly include phenols, carotenoids, and vitamins.

The largest part of antimicrobials derived from plants is contained in herbs and spices. These molecules exhibit multiple defensive mechanisms of action against foodborne pathogens, and the effects of structural variations of plant-derived components on their antimicrobial properties have been reviewed [62,63,64]. Due to the commitment of several research groups worldwide, the library of antimicrobial phytochemicals has been further enriched with thousands of compounds able to inhibit a wide range of microorganisms in vitro.

The main antibacterial phytochemicals are included in the principal categories of plant-derived phytochemicals. Hence, many natural compounds demonstrate enhanced effectiveness due to the simultaneous combination of both antioxidant and antimicrobial activities [65]. 

In addition to considering the biological activity of natural substances, it is also important to take into account the wide heterogeneity in the composition of plant extracts; each compound, in fact, displays peculiar characteristics and may contribute with a synergistic, antagonistic, or no effect to the biological outcome through different mechanisms. For example, spice and herb-derived essential oils (EOs), a mixture of natural volatile compounds, revealed antibacterial and antioxidant properties in animals as well as in humans [66]. 

Another driving factor for the renewed interest in plant antioxidant/antimicrobial applications in the past 20 years has been the rapid rate of plant species extraction technologies.

### 3.2. Innovative Extraction Technologies from Natural Sources

In recent years, innovative technologies have allowed the industrialization of green processes and large-scale diffusion of products based on natural matrices, which can guarantee lower environmental and economic impacts, reducing the loss of biologically active compounds without increasing extraction time.

In this context, the processes for the extraction and purification of natural AIs and their conversion into semi-finished and finished products should be as sustainable and green as possible [67]. 

The choice of the proper methodology is crucially driven by the physical-chemical characteristics of the bioactive molecules to recover. Natural compounds are often unstable at high temperatures or different pH values, and their organoleptic properties are significantly affected by the extraction conditions [68]. The availability of raw material(s), other integrated processes and their versatility, and the availability of workers, chemicals, and energy sources in a specific territory are also important factors for upscaling the extraction processes [68,69,70].

The main innovative techniques for the green extraction of natural bioactive principles are microwave-assisted extraction (MAE), ultrasound-assisted extraction (UAE), and supercritical fluid extraction (SFE), eventually in combination with solvent extraction and/or enzymatic treatment [71,72,73,74]. If necessary, purification and pre-concentration steps are usually performed by membrane separation and reverse osmosis techniques [75,76,77], and vacuum concentration and spray drying are two possible final procedures to obtain concentrated solutions or dried powders [74,78,79,80].

MAE has attracted the attention of researchers as a technique able to efficiently extract bioactive compounds from a wide variety of plants and natural residues. Microwaves produce electromagnetic radiation that occurs at frequencies between 300 MHz and 300 GHz and wavelengths between 1 cm and 1 m. The main advantage of this technique is to reduce the loss of extracted biochemical compounds [81], together with the optimization of time and solvent volume [82]. In detail, MAE is mainly used for heating the solvent and extracting antioxidants from plants with a smaller amount of chemicals [83]. 

UAE has been exploited in several applications of food processing technology to recover biologically active compounds from plant matrices. Ultrasound, with levels greater than 20 kHz, is utilized to disrupt plant cell walls, with the aim of improving the solvent capability to penetrate the cells and obtain a higher extraction yield. UAE relies on a low operating temperature through processing, maintaining a high extraction quality. For both this reason and the employment of common laboratory equipment (i.e., ultrasonic bath), UAE is considered one of the easiest extraction techniques. Practically, a smashed sample is mixed with the suitable solvent and placed into the ultrasonic bath under a controlled temperature and extraction time [84]. 

SFE represents one of the most promising technologies recently developed [84]. The definition of a supercritical fluid refers to the chemical at a temperature and pressure below its critical point (H_2_O: T_c_ = 374.15 °C, P_c_ = 218 atm; CO_2_: T_c_ = 31.1 °C, P_c_ = 72.8 atm). The choice of the subcritical fluid is crucial for the success of the extraction of bioactive compounds related to their chemical properties, as the polarity of supercritical water allows for the efficient extraction of polar compounds and supercritical carbon dioxide (sCO_2_) for non-polar substances. SFE is a green technology that avoids the use of toxic organic solvents and allows extraction at low temperatures. In addition, SFE is a versatile technology for two reasons: (1) the modulation of the operating parameters affects the physical-chemical properties of the supercritical fluid as the dielectric constant, viscosity, surface tension, and the diffusion coefficient change influencing the efficiency of the extractive process; (2) the combination of SFE with eco-friendly co-solvents (e.g., ethanol) allows for extracts with different chemical compositions to be obtained [84]. 

As previously mentioned, EEC Regulation 450/2009 laid down specific rules for active materials that are in contact with food, including a list of the authorized materials and their requirements. Hence, natural AIs extracted from plant tissues and used to give the polymer an active function must also have suitable characteristics according to the EEC regulation; in particular, they must be safe for human health and keep food unaltered. Most of the AIs used for these materials show antioxidant and antimicrobial activities that can be exploited to improve the storage conditions of packaged food. They are individual substances or standardized plant extracts containing blends of active compounds. 

### 3.3. Phenols

**Figure 4:**
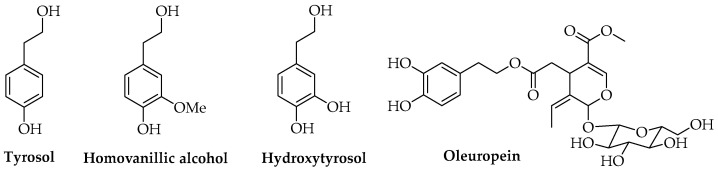
Phenolic compounds found in *Olea europaea* L.

At present, scientific studies are available in the literature concerning the use of antioxidant or antimicrobial natural compounds such as phenols, including both monomers and oligomeric or polymeric compounds, and terpenes. Antioxidant or antimicrobial hydrophilic phenols are reported as AIs for food applications and are extracted from different natural matrices. Bioactive compounds found in *Olea europaea* L., such as tyrosol, homovanillyl alcohol, hydroxytyrosol, and oleuropein, have been investigated (Figure 4), and promising results concerning the use of these molecules in active packaging polymer-based materials have been achieved [22,85,86].

Both commercial tyrosol and natural extracts of olive mill wastewater were tested on a laboratory scale by different research groups using antioxidant PLA-based films with suitable mechanical and chemical properties; in particular, their radical scavenging capacity, thermal enhancement, and stabilization effects for meat-derived foods and fruits were verified [87,88]. The use of olive wastewater was performed according to previous results concerning the exploitation of olive waste and by-products from a circular economy and sustainability perspective, thanks to their content in secondary metabolites. Olive mill wastewaters, namely the aqueous residues obtained in a three-phase oil extraction system, are rich in water-soluble antioxidant and bioactive principles such as hydroxytyrosol, phenolic compounds, polyalcohols, and contain lower amounts of lipids and pectins [88,89,90]. Oleuropein extracted from olive leaves was tested for obtaining biodegradable active PLA films that showed antimicrobial activity toward *Staphylococcus aureus* [91]. Moreover, the described laboratory extraction performed using chloroform and methanol could be replaced in an industrial context with more eco-friendly and up-scalable methods, such as MAE, UAE, and SFE, coupled with membrane filtration systems that allow for medium to high yields of targeted compounds to be reached [22,79,92,93,94,95]. 

In accordance with the territoriality of the species, availability of biomass and content in antioxidants, and the water-acetone, aqueous, and hydroalcoholic extracts of leaves from different vegetal species such as *Abies nordmanniana* (Steven) Spach; *Thymus vulgaris* L., *Camellia sinensis* L., *Rosmarinus officinalis* L. and *Liquidambar orientalis* Mill., *Aesculus hippocastanum* L. and commercial phenolics, such as ferulic acid and tannic acid, were selected in other studies and different active PLA materials for food and biomedical sectors have been obtained [96,97]. Polymers activated with tannic acid have shown good potentialities for wound dressing given their antioxidant and antimicrobial activity toward Gram-positive and Gram-negative bacteria, mechanical performance, and eco-compatibility [98]. The total antioxidant activity of the extracts was previously evaluated by in vitro assays, and the performed tests showed that PLA-based films activated with these components are essential in preserving food by extending its shelf-life in the case of limited storability [97,99,100,101,102]. Additionally, in these studies, laboratory processes are described, but for some of the reported species, industrial extraction processes to produce standardized extracts are available. 

**Figure 5:**
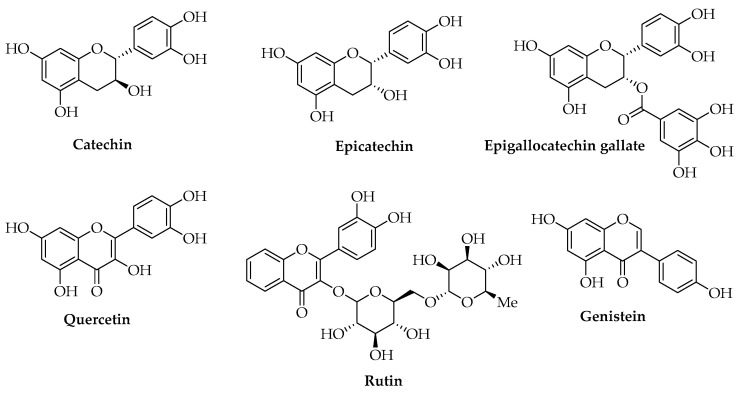
Chemical structures of the most common flavonoids.

*Camellia sinensis* L. leaves could represent another example of a vegetal raw material for which several large-scale processes are available with optimized parameters to produce extracts enriched in different compounds. The main phenols of green tea leaves are gallic acid, catechin, epicatechin, and epigallocatechin gallate (EGCG), with proven antioxidant and antimicrobial properties (Figure 5) [103]. Conventional solvent extraction methods using Soxhlet, maceration, reflux, or hydrodistillation have low efficiencies and often require high temperatures that lead to the degradation of thermolabile compounds. Moreover, they are expensive, time- and solvent-consuming, and in most cases, are not eco-friendly. To increase the yield of the antioxidant and antimicrobial fraction given by polyphenols, UAE allows for the application of lower temperatures, and the corresponding extract shows the best results in terms of antioxidant capacity [104,105,106]. Commercial catechin, epicatechin, EGCG, and other commercial phenols, such as quercetin, rutin, and genistein, have also been reported for obtaining PLA-based films with antioxidant and antimicrobial activity, good mechanical properties, and the ability to keep the quality of foods unaltered, not only on a lab-scale but also by using pilot-scale plants and green chemistry methodologies [97,107,108,109,110,111].

Some authors have produced an active PLA antimicrobial film for food packaging with a lab extract of *Allium ursinum* L. obtained by UAE of the bulbs in 70% ethanol to increase the yield of antimicrobial compounds, according to the results of previous studies concerning the chemical characterization of this species with high amounts of flavonoids and other phenols in addition to sulfur compounds [112,113].

**Figure 6:**
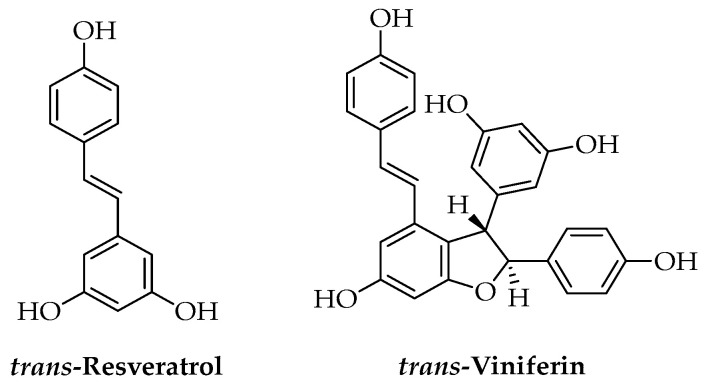
Chemical structure of the most important stilbenes.

Grapevine cane from *Vitis vinifera* L. pruning is an interesting by-product of viticulture that represents a valuable raw material containing high amounts of phenolic compounds; in particular, stilbenes such as *trans*-resveratrol and *trans*-viniferin, that could not be recovered from grape pomace (Figure 6). These compounds have a key role for *Vitis* in the defense against fungal pathogens and showed high antifungal activity both in vitro and in vivo towards different plant pathogens. Extracts from grapevine cane rich in stilbenes and other polyphenols were prepared using different methodologies: maceration in water or ethanol/water at 80 °C allowed high yields that decreased with long extraction times due to the thermal degradation of the compounds [114]. For large-scale extraction of grapevine cane, other technologies were described, including MAE, UAE, and SFE, which allowed efficient extraction yield for stilbenes; however, considering the yields, costs, and complexity of processes and plants, hydroalcoholic maceration remains a competitive extraction method [115,116]. PLA-based films, useful as sustainable food packaging materials, were produced with a hydroalcoholic grapevine extract obtained by maceration at 80 °C and then characterized for their physical-chemical properties. Unfortunately, the laboratory test showed low antifungal activity, probably due to an insufficient release of active principles, but the anti-adhesion tests revealed high effects preventing the adhesion of different bacteria, fungi, and yeasts when compared to polyethylene terephthalate and polystyrene [117].

Antioxidant and antimicrobial PLA-based materials were also prepared using phenolic polymers such as lignins and procyanidins. According to the available studies in the literature, both commercial lignin and lignin extracted from the tissues of different vegetal species such as *Saccharum munja* Roxb., *Triticum* sp., *Picea abies* L., and *Oryza sativa* L. [118,119,120,121,122,123] and from many biomasses (bamboo, pine, eucalyptus, spruce, and red maple) is available [124]. Lignin is not soluble in water, and it is often not miscible with PLA, so it is important to check its distribution inside the matrix during the processing of the active polymer. The polymerization degree is highly variable and different fractions, according to the average molecular weight, can be extracted and prepared with different techniques [118,124,125,126]. Industrial lignin is reported as a by-product of paper production and is currently used in a biorefinery context to produce bioethanol and energy, but it is furtherly exploitable for its antioxidant properties [118]. The enzymatic industrial extraction of procyanidins from hard matrices such as wood, peels, or seeds is very effective with respect to conventional solvent-based extractions, allowing for lower extraction times and temperatures. Furthermore, it makes it possible to standardize the obtained compounds in terms of yields and molecular weight using specific enzymes or mixtures of them and applying different process parameters such as temperature, time, concentration, and pH [72].

**Figure 7:**
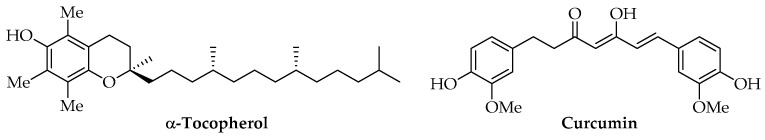
Chemical structure of α-tocopherol and curcumin.

Among lipidic phenols, α-tocopherol is the most investigated compound for producing PLA-based materials due to its high antioxidant properties and absence of toxicity at the tested concentrations (Figure 7). It is one of the most biologically important isoforms of vitamin E and a powerful lipid-soluble antioxidant [127]. Biodegradable antioxidant films for food packaging or microencapsulation systems for drugs were produced using commercial α-tocopherol [102,128,129,130] or samples extracted from *Galanthus elwesii* (snowdrop) leaves [97]. Many natural sources of this vitamin are available for food, cosmetic, and biomedical applications. It is present in high amounts in vegetable oils and wheat germs, even if natural extracts may be difficult to be standardized due to the variability of their concentrations. To overcome these difficulties, many synthetic methods have been optimized to use pure α-tocopherol [131].

Curcuminoids, such as curcumin (Figure 7), show antioxidant, anti-cancer, antibacterial, anti-inflammatory, and hypolipidemic activity [132]. Natural extracts have been obtained from *Curcuma longa* L. and used as food additives, colorants, or preservatives since the last century. Currently, the use of curcumin is considered neither economically nor environmentally sustainable due to its low concentrations in *Curcuma longa* L. rhizomes and the high request for solvent and energy for its extraction. Given its insolubility in water, SWE was tested as a green method at a laboratory scale with good yields; however, other production methods are also under study, mainly by engineered *Escherichia coli* [133]. Commercial curcumin was used for the preparation of active PLA films for food packaging with antioxidant, antimicrobial, and UV-screening activity and also tested for different concentrations of AIs [134,135]. Moreover, PLA/curcumin encapsulated systems and nanofilms were tested as innovative drug delivery systems and for other biomedical uses [136,137,138].

### 3.4. Terpenes

**Figure 8:**
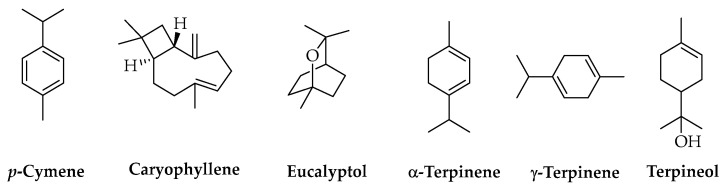
Chemical structures of terpenes.

Another important class of natural compounds studied as AIs for PLA-based materials is represented by terpenes. These include many structurally different molecules from the above-mentioned ones, and their main natural sources are the essential oils (EOs) produced by plants. Most of these compounds are characterized by high volatility and low chemical stability, which must be considered for selecting and optimizing extraction methods such as conventional distillation and SFE. PLA materials with terpenes as AIs are useful in the food and biomedical sectors due to the antimicrobial effects of these compounds. A crucial factor is given by the possible toxicity of some terpenes and their capacity to alter the organoleptic properties of food [139], so the assessment of AI amounts released into food and other matrices in contact with the polymer and their effects becomes particularly important. The literature reports studies performed using individual compounds or EOs obtained from different vegetable sources. Extracts from *Melaleuca alternifolia* (Australian tea tree) and *Leptospermum scoparium* (J.R.Forst. & G.Forst., 1776) (New Zealand manuka), respectively rich in *p*-cymene, caryophyllene, eucalyptol, α-terpinene, γ-terpinene, and terpineol (Figure 8), were used to prepare PLA fibers for biomedical applications [140], whereas *Cymbopogon citratus* (DC.) Stapf (lemongrass) EO, extracted by the steam distillation of dry leaves, was stabilized in PLA nanocapsules allowing for its use in the formulation of natural products against pathogenic fungi for green agriculture [141]. 

**Figure 9:**
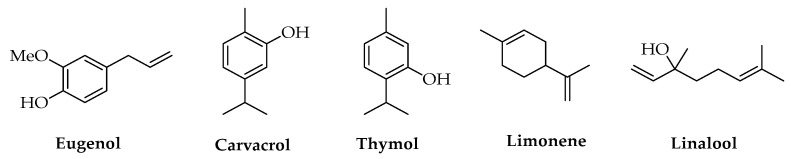
Chemical structures of terpenes.

Compounds such as eugenol, carvacrol, thymol, limonene, geranial and linalool (Figure 9) were used to prepare active food packaging materials based on PLA with antioxidant and antimicrobial properties to extend the shelf-life of products [142,143,144,145,146,147,148,149,150]. The versatility of SFE to obtain EOs and terpenes offered a chance to design productive processes of active PLA materials activated with individual terpenes or their mixtures that are up-scalable to obtain green and sustainable industrial processes. Supercritical impregnation using CO_2_ is a technique that allows for the enhancement of loading processes for AIs into different polymeric materials, including PLA, in terms of yields, preservation of the active compounds from evaporation/degradation, and physical-chemical characteristics of the obtained polymers [139,151,152]. It is possible to modulate the mechanical properties of the active polymers by modifying some parameters such as CO_2_ pressure, temperature, and contact time; in addition, for EOs/polymer material production, it is possible to couple SFE and impregnation steps to implement an integrated process, which is an advantageous perspective for industrial applications. These systems could be useful to minimize requests for time, energy, and equipment and avoid extract losses [151,153]. Among terpenes, thymol shows a strong antioxidant effect besides its antimicrobial activity [97,154]. Its main source is *Thymus vulgaris* L., from which it can be obtained by solvent extraction [97] or by SFE with CO_2_ as a mixture with other terpenes such as carvacrol, borneol, camphor, and 1,8-cineole [151,155]; otherwise, it can also be obtained from *Ziziphora clinopodioides* Lam. together with other active molecules such as carvacrol [156]. *Piper nigrum* L. (black pepper) is a source of antimicrobial and antioxidant terpenes such as β-caryophyllene, limonene, and α- and β-pinene that are extractable with sCO_2_ and useful to activate PLA-based polymers and confer them particular morphologies, allowing the production of materials suitable for biomedical applications [155]. Other aromatic plants, typically exploited for their content in EOs and reported for the activation of PLA, are *Origanum vulgare* L. [157,158], *Tanacetum balsamita* L. [159], *Salvia sclarea* L. [155,160], and *Zataria multiflora* Boiss. [161]. In some cases, natural extracts were used as AIs for PLA-based materials without reporting a detailed chemical characterization of the individual compounds present in the mixture; the quali-quantitative analysis of these extracts represents an interesting perspective of study to identify and concentrate or isolate the fractions responsible for the biological activity and possibly separate toxic compounds. 

**Figure 10:**
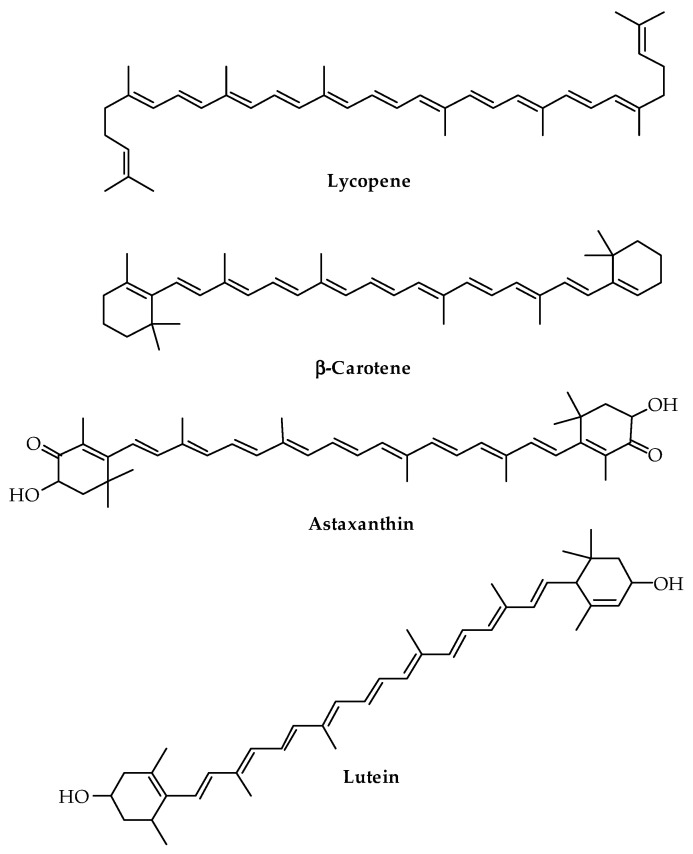
Chemical structure of the most important carotenoids.

Another important class of terpenes is carotenoids, largely used in feed, food, cosmetic, and pharmaceutical industries. In the literature, studies are reported concerning the activation of PLA-based polymers with lycopene, β-carotene, lutein, and astaxanthin (Figure 10), commercially available or extracted from vegetable matrices such as *Linum usitatissimum* L., *Tagetes erecta* L., and fruits of *Solanaceae*. Similarly, in this case, laboratory methods were tested to obtain antioxidant and antimicrobial active films or encapsulated systems for food packaging and pharmaceutical formulations with enhanced shelf-life [102,162,163,164,165]. Carotenoids are biological antioxidant principles synthesized not only by plant species but also by some bacteria and fungi exploitable for biotechnological industrial production [166]. Recent studies demonstrated the presence of genes encoding carotenoid biosynthetic enzymes in some insects, such as aphids and gall midges, possibly responsible for the production of pigments involved in color polymorphism [167]. For the industrial extraction of carotenoids, the most widespread methods are currently based on plant crops as sources, which have been improved by the available biotechnologies for enhancing the accumulation by plant species that naturally synthesize carotenoids or by activating their production as a new biosynthetic pathway if not naturally present [167]. The hydrophobic properties of carotenoids, their thermolability, and the specific content of metabolites in each natural source *versus* the targeted compounds are the main factors that determine the choice of pre-treatment, extraction, and purification methods. In addition to conventional methods, based on the use of high amounts of organic solvents, non-conventional methods such as SFE with CO_2_, UAE, pressurized liquid extraction, pulsed electric field extraction, and enzyme-assisted extraction were tested as non-thermal sustainable methods for large-scale extraction of carotenoids [168].

## 4. Active PLA-Based Materials

### 4.1. Processing Techniques

The release of active compounds from PLA films can be affected by the polymer processing method, so particular attention needs to be given to the different suitable methodologies for their incorporation, given that two main approaches are usually considered for the preparation of PLA-based materials: thermal processing (melt blending, compression molding, extrusion) and solvent-based procedures [169]. The limits of extrusion and solvent casting are clearly known, considering the disadvantages of solvent evaporation and the limited stability of AIs towards temperature. On the other hand, thermal processing is widely considered even at a scale superior to a laboratory scale, while solvent casting can be limitedly used for coatings. To preserve the activity of the incorporated AIs during the processing steps and provide the possibility of controlled release and targeted delivery, research aimed to mitigate their volatility, low solubility, and sensitivity to oxidation by different methodologies.

In the present review, for each AI reported in Table 1 and Table 2, plant materials (when available); incorporation technique (extrusion, electrospraying, electrospinning, encapsulation in nanocarriers, melt blending, solvent casting, sCO_2_ impregnation); characterization; antioxidant/antimicrobial activity; and food/biomedical application of the corresponding PLA-based materials are discussed.

#### 4.1.1. Encapsulation

The encapsulation of AIs in a carrier material is essential for many applications to prevent incompatibility between different matrices. Additionally, this technique provides protection by separating the AIs from the environment, preserving their chemical, physical, and biological properties. In the process of encapsulation, bioactive material, defined as the core, is coated with a single or combined material, named the shell or carrier. The core/shell structure can vary in shape and material selection depending on the applications and the requirements for specific release or delivery conditions (temperature, moisture) in different environments.

In this context, it is imperative to select suitable shell materials compatible with the properties of the core material in parallel to the choice of proper conditions of encapsulation and film formation. Commonly, nanoencapsulation is the favored pathway due to the possibility of having a larger surface of interaction with food and, thus, a higher opportunity for diffusion into the cell membrane of microorganisms. The inclusion of nanoencapsulated AIs into film packaging not only extends food storage times by preventing the growth of pathogens but also helps reduce the amounts of antimicrobial agents in direct contact with food products [170]. Many techniques, such as coacervation, nanoprecipitation, spray-drying, emulsification-solvent evaporation, ionic gelation, and electrochemical-based technologies, for the nanoencapsulation of bioactive compounds are available [171]. In recent years, several studies have highlighted the efficacy of electrospinning and electrospraying over the considered traditional encapsulation techniques [172,173,174]. Furthermore, these techniques are considered convenient due to the absence of heat for the thermal preservation of bioactive substances, and they are able to achieve high encapsulation efficiency values, ensuring the stability of compounds upon processing.

**Figure 11:**
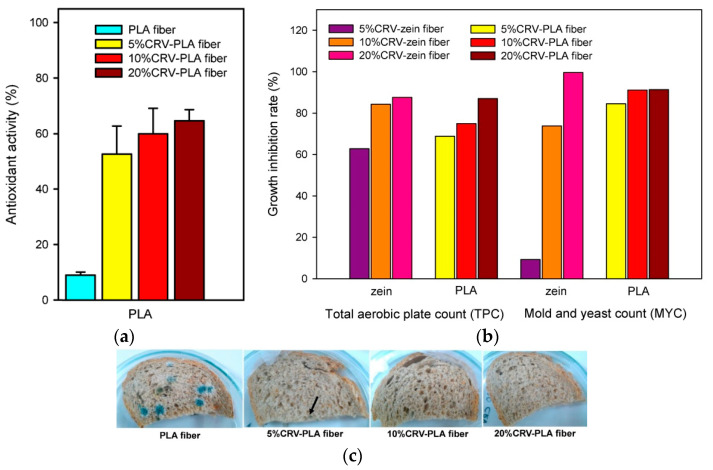
Antioxidant activity (%) of electrospun fibers of PLA with different carvacrol content (**a**); growth inhibition rate (%) of aerobic bacteria and mold and yeast of zein and PLA electrospun fibers (**b**); and images of whole wheat bread samples packed with electrospun PLA fibers with different carvacrol content stored at 25 °C for 7 days (**c**). Reprinted with permission from Ref. [144], Copyright 2018 Elsevier Ltd.

The direct encapsulation of AIs into the PLA matrix has also been explored. As an example, some authors have developed electrospun fibers of PLA combining carvacrol at different concentrations (5, 10, and 20%) [144]. They observed that the size and morphology of the fibers were affected by the amount of carvacrol, and preliminary studies on their application in whole wheat bread samples showed that encapsulation improved the monitored release of this specific AI (Figure 11). The results confirmed that 99.6% of mold growth was inhibited by 20% carvacrol content. In the paper by Min et al., it was proven that humidity-responsive PLA/PVA/PEG packaging films activated with thyme EO could be successfully realized by combining electrospinning and soaking. In vitro release behaviors of thyme EO from films were tuned by adjusting the humidity (20% RH to 80% RH), confirming the noticeable role of nanodimension in tuning film functional properties [175].

**Figure 12:**
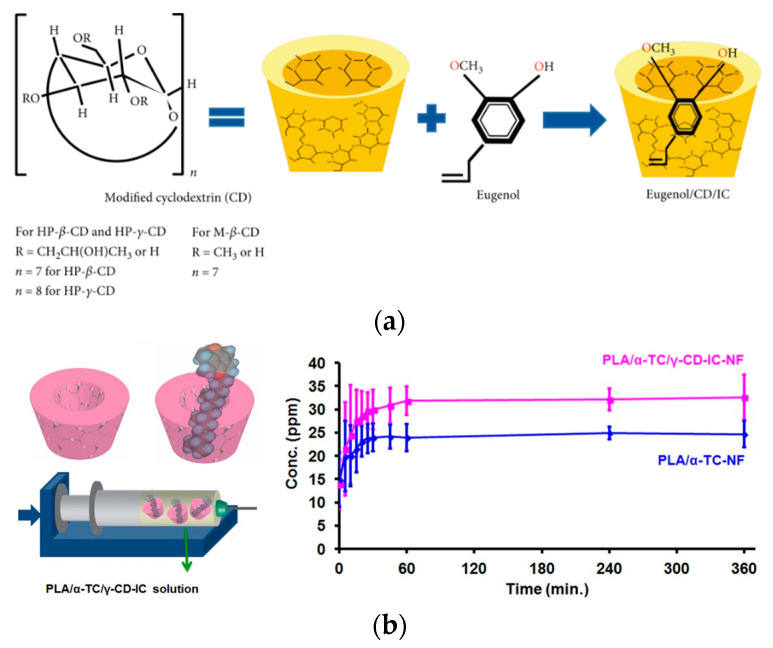
(**a**) Chemical structure of modified CD, eugenol, and schematic illustration of IC formation between CD and eugenol; Reprinted with permission from Ref. [178], Copyright 2020 Shahla Ataei et al. (**b**) schematic representations of γ-CD and α-TC/γ-CD–IC, electrospinning of nanofibers from a PLA/α-TC/γ-CD–IC solution and cumulative release of α-TC from PLA/α-TC–NF and PLA/α-TC/γ-CD–IC–NF into 95% ethanol. Reprinted with permission from Ref. [187], Copyright 2017 Wiley Periodicals, Inc., [187].

Different nanocarriers can also be incorporated into packaging materials [176,177] to modulate the release of the AI. It should be taken into account that the choice depends on both the food and the nanoparticles’ nature (Figure 12a) [178].

Among different strategies able to protect the AIs from thermal degradation, immobilization into a host solid support is considered a suitable approach. AIs have been successfully grafted on nanosilica, organo-modified montmorillonite, carbon nanotubes, and layered double hydroxides (LDHs). Among them, LDHs represent an excellent host matrix for the storage and delivery of bioactive molecules such as gallic acid and vanillic acid [179]. Their successful use has also been verified for PLA-based matrices: in the paper by Perez Amaro et al., the authors found that AIs-LDHs hybrids had a low tendency to migrate compared to AIs within PLA due to a robust ionic anchoring of AIs between the LDH layers [180]. 

Cyclodextrins (CDs) have shown to be exceptional carriers for AIs when directly included or complexed with nanosized fibers/particles. The assembly of cyclodextrin inclusion complexes (CICs) has been widely considered and is available in the literature [181,182]. Several research groups have already developed biodegradable packaging with promising functional properties [183]. For example, the preparation of an antimicrobial PLA-based material incorporating thymol and carvacrol complexes into β-cyclodextrin by means of an injection technique has recently been reported [184]. In the work of Velasquez-Contreras, PLA packages with thymol or carvacrol complexed in β-CDs were prepared to be employed as a preservation medium for berries, extending their shelf-life. PLA containing 5% β-CD-thymol showed a higher inhibition towards yeasts and molds (51.57%), with the berries still fit for consumption [185].

The combined use of CDs and the electrospinning methodology have also been considered and reported in the literature. As an example, α-tocopherol was encapsulated into γ-CD and combined with PLA into nanofibers by electrospinning, showing a faster release rate, due to the improved tocopherol solubility in the aqueous phase of the food simulant because to the presence of CD (Figure 12b) [186,187].

Wen et al. reported the use of PLA and complexed cinnamon EO (CEO) into β-CD [188]. CEO/β-CD was successfully obtained through the coprecipitation method, and then the complex was encapsulated into PLA nanofibers by electrospinning. The experimental results confirmed that the electrospun PLA/CEO/β-CD nanofilm exhibited better antimicrobial activity against *Escherichia coli* and *Staphylococcus aureus* than the PLA/CEO nanofilm. 

Oregano (EO) was complexed with β-CD and encapsulated in PLA/PCL electrospun nanofibers [189]. The authors demonstrated that the postharvest quality of blackberry fruit during storage under 25 °C and 55% RH was preserved for 4 days due to the effective barrier of the nanofibers toward moisture [189]. EO/β-CD/PLA/PCL nanofibers delayed the release of EO, contributing to long-term storage even at different temperatures. Moreover, hydrophobic molecules such as EOs can be loaded in nanoparticles based on different natural polymers (alginate, cellulose, chitosan, starch, etc.) using emulsification-solvent evaporation [190] and supercritical fluid technologies [191] that will be reviewed in the following section.

#### 4.1.2. Emulsification-Solvent Evaporation and sCO_2_ Impregnation

Encapsulation by emulsion-solvent evaporation consists of soaking the polymer solution into an emulsion, resulting in oil micelles involvement, and precipitation as capsules [192]. Unfortunately, when we consider PLA microcapsules containing AIs, not many papers are available, so this can be considered an enormous opportunity to search for this type of microcarrier’s behavior [193]. Capsules of PLA containing quercetin were prepared with an encapsulation efficiency of 96.7% and a loading of 19.4%. Release kinetics showed a biphasic release profile: 40%–45% of quercetin was released within 0.5 h and 87.6% released at 96 h, making these nanoparticles suitable candidates for nanomedicine [194]. In vitro studies demonstrated that nanocapsules of lemongrass EO and PLA show noteworthy antimicrobial activity and reduce biofilm formation in many clinically relevant microbial strains [195]. They can also be combined again with the electrospraying technique to prepare functional nanocapsules via coaxial electrohydrodynamic atomization. Nanocapsules were produced by combining PLA and plant extract of *Plumbago europaea* L. by selecting different extract concentrations and several flow rates. The investigation revealed that nanocapsules have a smooth spherical core-shell morphology with a fairly narrow size distribution and that bigger and monosized nanocapsules were formed only at high flow rates [196].

At present, there is an increasing demand for greener technologies to combine AIs and polymeric matrices, for example, supercritical impregnation using sCO_2_. The efficiency of the process depends on the polymer properties, chemical interactions between the AI and supercritical fluid, as well as the impregnation time and depressurization rate [197]. Villegas et al. impregnated thymol and cinnamaldehyde using sCO_2_ to test the structural, thermal, antimicrobial, and disintegration properties of PLA and PLA organoclay-containing films. Experimental data demonstrated that this approach represents a promising way to obtain active bionanocomposite films [139]. Rojas et al. recently showed that microcellular nanocomposite foams functionalized with cinnamaldehyde could be obtained through supercritical foaming, followed by an impregnation step, confirming the high potential of this technique for the migration and controlled release of AIs [198].

### 4.2. Properties

AIs incorporated into PLA films can improve not only functional performances, such as antioxidant and antimicrobial properties, but the inclusion of plant EOs, for example, may also improve the flexibility of the biopolymeric film due to their plasticizing effect, strongly correlated to type and concentration of AI and compatibility with polymer matrix [199].

Geranial and cinnamaldehyde are AIs added to PLA for their antibacterial activities. At the same time, they can improve barrier properties by modifying moisture content and water vapor permeability [200]. On the other hand, the microstructure of polymeric films combined with AIs could show coalescence, creaming, and droplet flocculation, which could happen during drying in the case of solvent casting or roughness on the film surface, contributing to considerably affect the antibacterial properties of the materials [201]. The roughness, color, transparency, and gloss of the packaging are highly influenced by the AI type and concentration. A slight color change, higher opacity, and reduced film transparency were observed for PLA-based materials prepared by incorporating rosemary, myrtle, and thyme EOs, and the effects were related to the type and concentration of EO [202]. 

Thermal properties of PLA can also be altered by processing conditions, AI molecular structure with its nucleating and/or plasticizing role, concentration, and degree of dispersion [198]. As expected, thermal behavior of active PLA produced through melting-based and solvent-cast processes are highly dependent on the temperature and solvent volatility, respectively. Release properties depend also on the crystallinity resulting from the different processing techniques. In line with this assumption, surface microstructure and wettability showed to be strongly related to chemical nature and amount of AI. In general, AIs able to plasticize PLA tend to accelerate PLA’s disintegration process, while AIs with nucleating properties tend to delay the disintegration step.

### 4.3. Antioxidant Activity

Evaluation of the antioxidant properties of active materials for food applications compared to neat matrices showed significant positive effects in a generally dose-dependent manner. Nevertheless, based on the qualitative and quantitative differences of used AIs, functionalization techniques, and final materials characteristics, it was not possible to perform a comprehensive analysis of the results. In general, PLA did not reveal antioxidant properties. However, a slight activity was recorded in a few works, probably related to LA residues [107,128,144,149,157].

Additionally, the antioxidant properties of natural compounds such as phenols and terpenes are strongly related to their chemical structure. Particularly, the presence of single or multiple hydroxyl groups in phenols is crucial because it allows these compounds to act as radical scavengers by donating hydrogen or electrons to intercept the oxidation chain and block ROS [203].

Regarding the assessment methods used for antioxidant properties evaluation, 2,2-diphenyl-1-picrylhydrazyl (DPPH) has been the most used. This colorimetric assay is based on the ability of an antioxidant to react with the stable free radical DPPH, resulting in the discoloration of the solution from an initial deep violet. The results were generally expressed as radical scavenging activity percentage (RSA %) at λ = 517 nm and compared with the control.

Monoterpenes, such as carvacrol and thymol, revealed antioxidant properties while activating PLA in several works using different functionalization methods. Ramos et al. tested the performance of PLA nanocomposite films, obtaining a 77.8 ± 0.1% DPPH scavenging activity [17]. Altan et al. investigated the ability of PLA/carvacrol fibers to react with the DPPH radical. The results showed that the significant differences in antioxidant properties of free AA carvacrol solutions depending on concentration were not confirmed in the electrospun fibers [144]. This is in accordance with Burgos et al., where no differences were recorded after DPPH tests of extracts from PLA-PHB film containing LA oligomers and carvacrol at different concentrations [143]. The relationship between the ability of natural compounds to fight oxidative stress and their structure not only depends on the quantitative presence of the “antioxidant active sites” but even on their distribution, which can lead to differences in results due to steric hindrance [204].

The antioxidant activities of materials for food applications have also been evaluated after migration processes and performed using an appropriate simulant, following the current legislation and standard methods [205,206].

The dynamic of the migration process, and consequently the results of the antioxidant evaluations, could be influenced by the physical-chemical properties of the materials, selectivity of the AI to the food simulant, temperature, and the capability of the hydroxyl group to establish hydrogen bonding with the polymeric matrix.

Flavonoids are one of the most represented classes of phenols in plants, especially as secondary metabolites. Rather than direct ROS scavenging, they exert their antioxidant activities by chelating metals and acting on red-ox enzymes. The maximum antioxidant potential of a flavonoid could be reached due to the simultaneous presence of a catechol structure in the B ring, hydroxyl groups in the C ring (3 and 5 position), and 4-oxo function in the C ring conjugated with a 2,3 double bond [203]. Quercetin includes all these criteria, resulting in a largely used compound in food applications. Ezati et al. investigated the antioxidant properties of the addition of quercetin on carboxymethyl cellulose (CMC), gelatin, and PLA films by DPPH and ABTS methods, revealing an enhanced effect even on UV-barrier properties [111].

A few works evaluated the overall flavonoid content by colorimetric assays in dried bulbs of *Allium ursinum* L. [113] and leaves of *Rosmarinus officinalis* L. [101] and *Camellia sinensis* L. [100]. The significative antioxidant power of polyphenols is strictly related to the presence of a catechol or pyrogallol moiety in the aromatic ring [203,207,208,209]. Iniguez-Franco et al. reported a positive antioxidant performance from PLA functionalized with catechin or epicatechin by extrusion [107]. The authors investigated the effects of the extraction temperature on the radical scavenging activities, evidencing a direct increase at 40 °C. DPPH tests performed on samples extracted at 50 °C revealed a reduction of the antioxidant effect, probably related to the thermal decomposition of catechin. In fact, some processing methodologies, such as extrusion, are carried out at high temperatures that could affect the antioxidant activities of AIs. However, few works reported an increase in the material performance compared to fresh extracts or commercial compound solutions, probably due to, as mentioned before, the slight radical scavenging activity of the PLA-based matrix. 

The use of phenolic polymers such as procyanidins and lignins on the PLA activation allows for the enhancement of both antioxidant and structural properties with a potential circular approach by re-using agro-industrial wastes such as seeds, leaves [97], husks [122], or straws [118].

Lipidic phenols combine the antioxidant properties of hydroxyl groups with the presence of conjugated double bonds along the aliphatic chain, which can contribute to free radicals scavenging. Cardanols from cashew nuts (*Anacardium occidentale* L.) were used as plasticizers by Mele et al. to develop, by extrusion, a film based on PLA with enhanced radical scavenging properties [210].

In the work of Munteanu et al., vitamin E (α-tocopherol) was used as the AI in PLA membranes through an electrospinning process [128]. The antioxidant potential of the active agent was enhanced by the presence of silver nanoparticles (Ag NP), in accordance with results from Asadi et al., where TiO_2_-supported lycopene pigments from tomato pulp provided the radical scavenging properties of a PLA-based film [164]. The extended chromophore chain in the lycopene structure increases its antioxidant potential, conferring a higher activity against single oxygen compared to α-tocopherol [211]. Most of the activation techniques allow the combination of two or more active substances and extracts, giving the opportunity to evaluate a synergistic effect. Lukic et al. revealed that the simultaneous addition of thymol and carvacrol to PLA revealed an increase in radical scavenging and a decrease in power activity [152]. These results partially agree with the findings revealed by Siddiqui et al. [149]. The authors, after assessing the antioxidant activities of PLA films activated with thymol, carvacrol, limonene, and cinnamaldehyde using the DPPH method, made comparisons between secondary, ternary, and quaternary blends. The results showed the highest antioxidant activities of the composite PLA/carvacrol [149].

**Table 1:** Phenolic compounds and polymers used as AIs for PLA-based materials.

Class	Compound	Source	AI Incorporation Technique	Activity	Application	Ref.
Curcuminoids	Curcumin	*Curcuma longa* L.	Solvent casting	Antioxidant Antimicrobial	Food	[134]
	Curcumin	*Curcuma longa* L.	Solvent casting	Antioxidant Antimicrobial	Food	[135]
	Curcumin	*Curcuma longa* L.	Electrospinning	Antioxidant	Biomedical	[136]
	Curcumin	*Curcuma longa* L.	Encapsulation	Antioxidant	Biomedical	[137]
	Curcumin	*Curcuma longa* L.	Emulsification	Antioxidant	Biomedical	[138]
			solvent evaporation	Antimicrobial		
Flavonoids		*Camellia sinensis* L. (powder)	Electrospinning	Antimicrobial	Food	[96]
		*Camellia sinensis* L. (leaves)	Extrusion	Antioxidant	Food	[100]
		*Rosmarinus officinalis* L. (leaves)	Melt mixing	Antioxidant Antimicrobial	Food	[101]
		*Allium ursinum* L. (dried bulbs)	Solvent casting	Antimicrobial	Food	[113]
	Catechin	*Abies Nordmanniana* (Steven) Spach (leaves)	Solvent casting	Antioxidant	Food	[97]
	Catechin	Commercial	Melt mixing	Antioxidant		[107]
	Catechin	Commercial	Electrospinning	Antioxidant	Food	[109]
				Antimicrobial		
	Epicatechin	*Liquidambar orientalis* Mill. (leaves)	Extrusion	Antimicrobial		[99]
	Epicatechin		Melt mixing	Antioxidant		[107]
	Epigallocatechin gallate	Commercial	Thermo-compression molding	Antioxidant Antimicrobial		[110]
	Genistein	Commercial	Electrospinning	Antioxidant		[108]
	Quercetin	Commercial	Solvent casting	Antioxidant Antimicrobial	Food	[111]
	Rutin	*Olea europaea* L. (leaves)	Encapsulation	Antioxidant Antimicrobial	Cosmetic	[86]
Phenethyl alcohols	Hydroxytyrosol	*Olea europaea* L. (by-products)	Solvent casting	Antioxidant	Food	[88]
Lipidic phenols	Cannabidiol	*Linum usitatissimum* L. (fibers)	Compression molding	Antioxidant Antimicrobial	Food	[162]
	Cardanols	*Anacardium occidentale* L. (oil)	Melt extrusion	Antioxidant	Food	[210]
	α-Tocopherol	Commercial	Electrospinning	Antioxidant Antimicrobial	Food	[128]
	α-Tocopherol	Commercial	Emulsion solvent evaporation	Antioxidant	Food	[129]
	α-Tocopherol	Commercial	Melt blending	Antioxidant	Food	[130]
Phenolic acids	4-Hydrobenzoic acid	*Linum usitatissimum* L.	Compression molding	Antioxidant Antimicrobial	Food	[162]
	Gallic acid	Commercial	Encapsulation Electrospinning	Antioxidant	Food	[186]
	Protocatechuic acid	*Linum usitatissimum* L. (leaves)	Extrusion	Antimicrobial	Food	[99]
	Rosmarinic acid	Commercial	Extrusion	Antioxidant Antimicrobial	Food	[17]
	Rosmarinic acid	Commercial	Encapsulation Supercritical emulsion extraction	Antioxidant	Biomedical	[102]
	Rosmarinic acid	Commercial	sCO_2_ impregnation	Antimicrobial	Food	[139]
	Rosmarinic acid	Commercial	Extrusion	Antimicrobial	Food	[142]
	Rosmarinic acid	Commercial	Melt blending	Antioxidant	Food	[145]
	Rosmarinic acid	Commercial	Impregnation	Antioxidant Antimicrobial	Food	[148]
	Rosmarinic acid	Commercial	Melt mixing Solvent casting	Antioxidant	Food	[149]
	Rosmarinic acid	Commercial	sCO_2_ impregnation	Antimicrobial	Food	[151]
	Rosmarinic acid	Commercial	sCO_2_ impregnation	Antioxidant	Food	[152]
	Tannic acid	Commercial	Layer-by-layer	Antioxidant Antimicrobial	Biomedical	[98]
Phenolic extract		*Abies Nordmanniana* (Steven) Spach (leaves)	Solvent casting	Antioxidant	Food	[97]
		*Aesculus hippocastanum* L. (seeds)	Solvent casting	Antioxidant	Food	[97]
		*Camellia sinensis* L. (leaves)	Extrusion	Antioxidant	Food	[100]
		*Rosmarinus officinalis* L. (leaves)	Extrusion	Antioxidant Antimicrobial	Food	[101]
		*Durvillaea antarctica* (Chamisso) Hariot (algae) (powder)	Solvent casting	Antioxidant Antimicrobial	Food	[109]
		*Allium ursinum* L. (bulb)	Solvent casting	Antimicrobial	Food	[113]
Phenolic polymers	Lignin	Commercial	Extrusion	Antioxidant	Food	[118]
	Lignin	Commercial	Solvent casting	Antioxidant	Biomedical	[119]
	Lignin	Commercial	Melt mixing	Antioxidant Antimicrobial	Biomedical	[120]
	Lignin	Commercial	Melt mixing	Antioxidant		[121]
	Lignin	Commercial	Solvent casting	Antioxidant	Food	[122]
	Lignin	Commercial	Melt mixing	Antioxidant	Food	[123]
	Melatonin	Commercial	Nanoprecipitation	Antioxidant	Biomedical	[212]
	Procyanidins	*Abies Nordmanniana* (Steven) Spach (leaves)	Solvent casting	Antioxidant	Food	[97]
	Procyanidins	*Aesculus hippocastanum* L. (seeds)	Solvent casting	Antioxidant	Food	[97]
Secoiridoids	Oleuropein	*Olea europaea* L.	Encapsulation	Antioxidant Antimicrobial	Cosmetic	[86]
	Oleuropein	*Olea europaea* L.	Solvent casting	Antimicrobial	Food	[91]
Stilbenes	trans-Resveratrol	*Vitis vinifera* L. (cane)	Melt mixing	Antimicrobial	Food	[117]
	trans-Viniferin	*Vitis vinifera* L. (cane)	Melt mixing	Antimicrobial	Food	[117]
Xanthonoids	Mangiferin	*Mangifera indica* L. (leaves)	sCO_2_ impregnation	Antioxidant	Biomedical	[213]

**Table 2:** Benzaldeydes and terpenes used as AIs for PLA-based materials.

Class	Compound	Source	AI Incorporation Methodology	Activity	Application	Ref.
Benzaldeydes	Cinnamaldeyde	Commercial	Impregnation	Antimicrobial	Food	[139]
	Cinnamaldeyde	Commercial	Melt mixing Solvent casting	Antioxidant	Food	[149]
	Vanillin	*Olea europaea* L. (leaves)	Encapsulation	Antioxidant Antimicrobial	Cosmetic	[86]
Carotenoids	Astaxanthin	*Tagetes erecta* L. (flowers)	Extrusion	Antioxidant	Food	[163]
	β-Carotene	Commercial	Encapsulation Supercritical emulsion extraction	Antioxidant	Biomedical	[102]
	Lycopene	*Solanum lycopersicum* L. (pulp)	Solvent casting	Antioxidant Antimicrobial	Food	[164]
	Lycopene	Commercial	Solvent casting	Antioxidant	Food	[165]
	Lutein	*Linum usitatissimum* L. (fibers)	Compression molding	Antioxidant Antimicrobial	Food	[162]
Terpenes	Abietic acid	*Abies Nordmanniana* (Steven) Spach (leaves)	Solvent casting	Antioxidant	Food	[97]
	β-Bisabolene	*Tanacetum balsamita* L.	Solvent casting	Antimicrobial	Food	[159]
	Carvacrol	Commercial	Extrusion	Antioxidant Antimicrobial	Food	[143]
	Carvacrol	Commercial	Electrospinning	Antioxidant Antimicrobial	Food	[144]
	Carvacrol	Commercial	Encapsulation	Antimicrobial	Food	[146]
	Carvacrol	Commercial	Electrospinning	Antioxidant Antimicrobial	Food	[147]
	Carvacrol	Commercial	Melt mixing Solvent casting	Antioxidant	Food	[149]
	Carvacrol	Commercial	sCO_2_ impregnation	Antioxidant	Food	[152]
	Carvacrol	*Ziziphora clinopodioides* Lam.	Solvent casting	Antioxidant Antimicrobial	Food	[156]
	Carvacrol	*Zataria multiflora* Boiss.	Solvent casting	Antimicrobial	Food	[161]
	β-Caryophyllene	*Piper nigrum* L.	Electrospinning	Antimicrobial	Biomedical	[160]
	Carvone	*Tanacetum balsamita* L.	Solvent casting	Antimicrobial	Food	[159]
	*p*-Cymene	*Leptospermum scoparium* (J.R.Forst. & G.Forst., 1776)	Electrospinning	Antimicrobial	Biomedical	[140]
	Eucalyptol	*Melaleuca alterniflora* (Maiden & Betche) Cheel, 1924 (oil)	Electrospinning	Antimicrobial	Biomedical	[140]
	Eucalyptol	Commercial	Flow-delay	Antioxidant Antimicrobial	Food	[150]
	Eugenol	Commercial	Impregnation	Antioxidant Antimicrobial	Food	[148]
	Geranial	*Cymbopogon citratus* (DC.) Stapf	Encapsulation	Antimicrobial	Food	[141]
	Limonene	Commercial	Melt mixing Solvent casting	Antioxidant	Food	[149]
	Limonene	*Piper nigrum* L.	Electrospinning	Antimicrobial	Biomedical	[160]
	Linalool	Commercial	Encapsulation	Antimicrobial	Food	[146]
	Linalool	*Salvia sclarea* L.	Electrospinning	Antimicrobial	Biomedical	[160]
	Linalool	*Zataria multiflora* Boiss.	Solvent casting	Antimicrobial	Food	[161]
	Linalyl acetate	*Salvia sclarea* L.	Electrospinning	Antimicrobial	Biomedical	[160]
	β-Myrcene	*Cymbopogon citratus* (DC.) Stapf	Encapsulation	Antimicrobial	Food	[141]
	Neral	*Cymbopogon citratus* (DC.) Stapf	Encapsulation	Antimicrobial	Food	[141]
	α-Pinene	*Piper nigrum* L.	Electrospinning	Antimicrobial	Biomedical	[160]
	β-Pinene	*Piper nigrum* L.	Electrospinning	Antimicrobial	Biomedical	[160]
	Sesquiterpenes	*Leptospermum scoparium* (J.R.Forst. & G.Forst., 1776)	Electrospinning	Antimicrobial	Biomedical	[140]
	α-Terpinene	*Melaleuca alterniflora* (Maiden & Betche) Cheel, 1924	Electrospinning	Antimicrobial	Biomedical	[140]
	γ-Terpinene	Commercial	Electrospinning	Antimicrobial	Biomedical	[140]
	Terpineol	*Melaleuca alterniflora* (Maiden & Betche) Cheel, 1924	Electrospinning	Antimicrobial	Biomedical	[140]
	Terpineol	*Salvia sclarea* L.	Electrospinning	Antimicrobial	Food	[160]
	Thymol	*Thymus vulgaris* L. (leaves)	Supercritical impregnation	Antimicrobial	Food	[151]
	Thymol	*Ziziphora clinopodioides* Lam.	Solvent casting	Antioxidant Antimicrobial	Food	[156]
	β-Thujone	Commercial	Electrospinning	Antioxidant Antimicrobial	Food	[96]
	β-Thujone	*Zingiber officinale* Roscoe	Melt blending	Antioxidant	Food	[130]
	β-Thujone	*Thymus vulgaris* L.	Solvent casting	Antimicrobial	Food	[154]
	β-Thujone	*Origanum vulgare* L.	Extrusion	Antioxidant Antimicrobial	Food	[158]
	β-Thujone	*Tanacetum balsamita* L.	Solvent casting	Antimicrobial	Food	[159]

The use of EOs could allow the functionalization of a polymeric matrix with a wide range of compounds in a faster and easier way. Nevertheless, although this approach may appear even more sustainable, the lack of reproducibility could pose a significant risk for the use of these active materials in the market. For an industrial scale-up and commercialization of PLA-AI materials, stability plays a crucial role. Chen et al. recorded an almost complete loss of the radical scavenging ability of PLA directly activated with eucalyptus leaf extract within 12 days [150]. On the other hand, the encapsulation of EOs in fish gelatin and corn starch before the film development revealed a significant delay in losses, which could open an elongated duration of antioxidant properties. The activation method could represent a key factor in keeping the antioxidant properties. sCO_2_ impregnation used by Lukic et al. to plasticize a PLA/PCL film with thymol and carvacrol revealed good antioxidant performance even after 1 month of storage [152].

Regarding the evaluation of the antioxidant properties of PLA-AI materials, the choice of the method is crucial and could lead to different interpretations of results. Chen et al. compared the DPPH results with those obtained by applying the ABTS (2,20-azino-bis(3-ethylbenzthiazoline-6-sulfonic acid) test, and experienced differences in the absolute values, even though the trends were similar [150]. This could be attributed to the selective efficiency of AI against test free radicals. Then, it could be interesting to use more than one method for a comprehensive and complementary approach.

In vitro methods are largely used and offer reliable results, even in reproducibility terms, with limited cost and time requirements. A few articles reported analysis performed on foods packed with PLA-AI materials. As expected, most assessments were borne by lipid oxidation. Lipids, in fact, represent one of the main classes of organic compounds involved in oxidative processes, which negatively affect the qualitative aspects of foods and their shelf-life. Peroxide value (PV) was one of the most performed analyses to evaluate the oxidation status of lipids. Shavisi et al. found a significant effect in the reduction of peroxides during the packaging of minced beef with PLA activated with ethanol extract of an EO from *Ziziphora clinopodioides* Lam., when compared to the control [156]. The results also showed a synergistic effect between the two AIs, allowing an extension of the meat shelf-life in refrigerated conditions of at least 11 days. In the work of Martins et al., the effects of a packaging composed of PLA and different green tea extracts on lipids of smoked salmon were evaluated using PV, thiobarbituric acid reactive substances (TBARS), hexanal assay, and *p*-anidisine value [100]. All methods provided a positive result with significant differences from the control. The presence of double bonds exposed lipids composed mainly of mono and polyunsaturated fatty acids to oxidative processes. Gas chromatography offers an alternative solution to colorimetric assays by testing the changes that occurred to the fatty acid profiles during storage. Some authors investigated the antioxidant properties of a double-layer PLA/gelatin/EGCG film obtained by compression molding in the packaging of striped catfish. A GC-FID analysis revealed a significant difference in the mono and polyunsaturated fatty acids content and composition of samples compared to a control packed with both PLA/gelatin and LLDPE, attributing beneficial performance only to the presence of EGCG [110].

Lipid degradation leads to the formation of unpleasant compounds that adversely affect the sensory characteristics of the food. Sensorial evaluation with a colorimeter was recorded on avocados [88] and salami [87], then the panel methodology was applied to catfish [110] and beef [156]. Jiang et al. evaluated the levels of oxidative enzymes in peaches packaged with a film in PLA/poly (3-hydroxybutyrate-co-4-hydroxybutyrate) activated by extrusion with ginger EO (*Zingiber officinale* Roscoe) and *Angelica acutiloba*, finding in both cases an inhibitory effect against the enzyme LOX [130]. Tocopherols are Generally Recommended as Safe (GRAS) AIs by the FDA and used as AIs in a PLA/poly (3-hydroxybutyrate-co-4-hydroxybutyrate) film by Jiang et al. in peaches storage revealing great firmness. Other determinations were performed, and changes occurring in terms of antioxidant levels could be detected by spectrophotometric assays and using chemical-physical evaluations, such as pH, acidity, weight loss, and water activity [130].

The use of degradable polymeric materials for the encapsulation of AIs can be an important solution in the field of drug delivery and nutrition. In the work of Gimenez-Rota et al., β-carotene, α-tocopherol, and rosmarinic acid were encapsulated in PLA/poly (lactic-co-glycolic acid) nanocarriers through supercritical emulsion extraction resulting in a synergistic effect on DPPH reduction. However, the results revealed a reduced encapsulation efficiency by rosmarinic acid compared to α-tocopherol and β-carotene. The developed system allowed for improved drug shelf-life for two years. Finally, the authors experienced the protective role of α-tocopherol on β-carotene during co-encapsulation [102].

Bioavailability and cytotoxicity represent two of the main weaknesses limiting the application of AIs in biomedical and pharmaceutical fields. In an in vitro study, PLA activated with melatonin significantly reduced the levels of ROS in the splenocytes of golden hamsters. The active material showed controlled release kinetics and efficacy comparable to pure melatonin and thermal properties such as neat PLA [212]. These results agreed with Kai et al., who prepared a PLA/lignin copolymer showing strong radical scavenging properties and good cytocompatibility against three different cell lines (PC12, human fibroblasts, and human mesenchymal stem cells) [119].

Lignins were also used as active agents in the development of PLA composite for 3D printing, which actually represents one of the most diffused commercial uses for this biopolymer. The printed material revealed good antioxidant properties even after the extrusion processes, according to the results of the DPPH method [120]. This work revealed the chance to use activated 3D-printed PLA even for healthcare applications such as wound healing. PLA 3D filaments were activated by mangiferin-rich mango leaves extracts through sCO_2_ impregnation [213] or curcumin [120]. Curcumin revealed great ability in the activation of antioxidant PLA-based materials through several functionalization methods, such as nanoprecipitation [137], emulsion [138], and electrospinning [136]. Despite the first being related to drug delivery applications, electrospinning represents the most interesting method for the development of wound care materials. Some authors investigated the effects of electrospun PDLLA/poly (ethylen oxide)/geninstein mats, revealing significative antioxidant and anti-inflammatory effects related to the polymeric structure and offering, at the same time, a tangible solution to overcome the limited bioavailability of genistein [108].

Other authors investigated the encapsulation of olive leaf extract in PLA nanoparticles for potential cosmetic use. The extract, rich in oleuropein and rutin, revealed promising antioxidant properties even after the encapsulation process, which conferred enhanced stability without affecting the technological and organoleptic properties [86].

### 4.4. Antimicrobial Activity

The design and development of antimicrobial packaging represent one of the most interesting challenges for academics and industries to prevent and control food spoilage. Due to their bioavailability and low toxicity for humans, AIs are suitable candidate weapons to fight against harmful microorganisms that affect food quality and safety [214]. The processes of food manufacturing are, in fact, subjected to contaminations which could represent a critical risk to the safety of citizens. In parallel, as previously commented, a commercial weakness for PLA-AI packaging development could be represented by the possibility of altering color and odor, when the AI is in contact with food.

The antimicrobial properties of terpenes are mainly due to their lipophilic character, resulting in an effect on the structure and functionality of microbial cell membranes [215]. The activation of PLA-based matrices with thymol revealed antimicrobial effects on the most important bacterial related to food-borne illness, such as Gram-negative *Escherichia coli* [17,139,145,148,151], *Pseudomonas* spp. [142], and Gram-positive *Bacillus subtilis* [151], *Staphylococcus aureus* [17,139,145], and *Micrococcus lysodeiktus* [142].

The use of oregano EO is largely diffused as a preservative agent in the food industry due to its antioxidant and antimicrobial properties mainly related to a high monoterpenes content [216]. Some authors revealed the potential of this AI for PLA-based materials in antimicrobial films with in vitro activity against *Enterococcus faecalis*, *Listeria monocytogenes*, *Staphylococcus aureus*, *Staphylococcus carnosus*, and *Yersinia enterocolitica*. Moreover, the authors evidenced an effect on the growth of yeasts and molds in ready-to-eat salads using a PLA film activated with 5% and 10% of EO [158]. These results are in agreement with the experiment performed by Liu et al., where oregano EO was used to activate PLA/poly(thrimethylene carbonate) by solvent casting. The incorporation of a 9% wt. of EO provided strong antimicrobial activity against *Escherichia coli* and *Listeria monocytogenes* combining simultaneously good mechanical, thermal, and barrier properties [157]. For the development of antimicrobial PLA-based materials, a few terpenes-rich EOs were obtained from *Zataria multiflora* Boiss. [161], *Tanacetum balsamita* L. [159], *Thymus vulgaris* L. [154], and *Salvia sclarea* L. [160].

The antimicrobial effects of terpenes could be exerted even as alternatives to fungicides. Some authors investigated the effects of lemongrass EO (*Cymbopogon citratus* (DC.) Stapf) nanoencapsulated in a PLA matrix against pathogenic fungi. In vitro results showed a MIC of 0,1% (*v*/*v*) against *Colletotrichum acutatum* and *Colletotrichum gloeosporioides*. The antimicrobial activity was confirmed by in vivo tests on harvested apples resulting in three times smaller bitter rot lesions compared to the control [141]. Another feature of terpenes on the application as active agents is due to their general volatility. This could allow them to be used even in the development of packaging that exerts antimicrobial activity without direct contact with food.

Roy et al. evaluated the effect of a composite PLA/curcumin film produced by solvent casting against both Gram-negative (*E. coli*) and Gram-positive (*L. monocytogenes*), revealing a reduction of 1-2 log cycle of the viable colony compared to neat PLA [135]. The positive effects of PLA/curcumin film on E. coli growth inhibition were evidenced even by Hanafi et al. However, the authors tested the antimicrobial properties against *Bacillus cereus* and *Pestalutiopsis*, *Penicillium*, and *Aspergillus niger* fungi, revealing a weak and vain effect, respectively, probably due to the different membrane composition of Gram-positive bacteria and fungi, which generates trouble to curcumin permeation [134].

The preventive role of flavonoids against microorganisms revealed on plants and humans offers a hint at the development of antimicrobial active packaging. Quercetin could be a key representative, even for the status of GRAS, according to the FDA. Ezati et al. investigated the properties of quercetin on the activation of PLA, gelatin, and CMC matrix against *E. coli* and *L. monocytogenes*. The results agreed with the trend registered for curcumin, observing higher effects on the Gram-negative than the Gram-positive bacteria but revealed a reduced effect of the PLA-based system compared to the others due to a small release rate of quercetin [111].

The contribution of a circular approach to the development of active bio-based packaging could be enhanced using AIs from agro-industrial wastes. Erdohan et al. used an oleuropein-rich leaf extract from *Olea europaea* L. as an active agent for the development of a PLA-based film with antimicrobial activity towards *S. aureus* in vitro cultures [91]. 

Diaz-Galindo et al. demonstrated the possibility of obtaining an antimicrobial PLA film using grapevine cane extracts rich in trans-resveratrol and trans-viniferin. The film revealed antifungal effects against *Botrytis cinerea* in a directly proportional manner and no effect on *Listeria monocytogenes*, *Saccharomyces pastorianus*, *Pseudomonas aeruginosa,* and *Pectobacterium carotovorum* [117].

These works highlighted the troubles related to the migration of phenolic compounds from the PLA matrix, which can partially or totally affect the results of antimicrobial tests. The use of different methods makes it even more difficult to make comparisons on the real potential of materials, as they can provide different results even with the same materials and targets. The use of recognized and accredited methods represents a fundamental step for the enhanced comparability of results and mainly for the compliance of the active material with national, community, and international standards, which represent a basic pre-requirement for commercial use. Among these methods, there are some specific for the evaluation of the antimicrobic activity of polymer matrices, such as ASTM E2180 and JIS Z2801/ISO 22196 [217]. These are very similar methods that allow a quantitative determination through direct exposure of the sample and control by direct contact with solutions of a known concentration of reference bacteria for a defined time, usually 24h. At the end of the exposure phase, the bacteria are recovered and counted. The antimicrobial activity of the polymeric matrix is generally expressed as a percentage change or logarithmic reduction of the bacterial concentration compared to the initial one [218].

Masek et al. used the ASTM E2180 method to evaluate the antimicrobial activity of a PLA matrix activated through an impregnation process with commercial thymol, eugenol, and *Thymus vulgaris* L. extracts. All formulations showed a reduction of E. coli colonies, but the composite PLA/thymus extract achieved the best performance [148].

PLA was activated with *Allium ursinum* L. extracts by solution casting, revealing significant antimicrobial activities with 0.5% wt. presence of the active agent [113]. The standard method used is comparable with ISO 22196 applied by Łopusiewicz et al., where an effect on *Enterococcus faecalis*, *Pseudomonas putida*, and *P. aeruginosa* was recorded using an extruded film based on PLA and melanin from *Agaricus bisporus* wastes. However, it should be borne in mind that authors generally use similar methods to the above-mentioned official standards with some variations, for example, by assessing the adhesion of bacterial cells to the surface [117] or simulating artificial contamination [113].

The antimicrobial activities could be assessed even with dynamic contact-based methods, e.g., using the reference ASTM 2149 method [109,139]. This procedure, specific for immobilized antimicrobial agents, is very similar to the previous ones, with the difference being the inoculum is placed in contact with the material on the bottom of the flask and stirred for a defined time. For this reason, while the direct contact-based methods simulate the development of a “pseudo-biofilm”, the stirring phase promotes a greater interaction. For this reason, the ASTM 2149 method is also suitable for tests on nanomaterials and nanofibers. Liu et al. evaluated the antimicrobial activity of electrospun nanofibers made by PLA and tea polyphenols through an increased vibration method, which essentially combines the two types of above-mentioned methods through a first phase of static direct contact and a subsequent one with action. The results on tested *S. aureus* and *E. coli* were promising, and no activity was shown by the neat PLA, which allowed for the attribution of the gain of antimicrobial properties exclusively to the active agent [96]. Among works that mentioned official methods, many of them referred to the identification of bacterial species. These could be based on serial dilution and colony counts, like the SR ISO 16649 specific for *E. coli* [101,128]. The CLSI standard M07 [143,146,159] or NCCLS M2-A8 are based on agar disk diffusion [91]. This type of analysis, largely diffused, was developed in the 1940s to evaluate the antibiotic effect of penicillin. It consists of placing the tested material in the center of a plate where the bacterial targets have been inoculated at a defined concentration. The antimicrobial effect of the active material is diagnosed by the formation of a halo in a direct correlation manner [219]. Milovanovic et al. evaluated the antimicrobial activity of a film consisting of a blend of PLA and 5% poly-caprolactone functionalized with thymol and thyme extracts using sCO_2_ impregnation and a bioluminescence assay for ATP. This method evaluates a bacterial suspension placed in contact with the reference material and the levels of ATP, linearly related to proliferation, by measuring the optical density at l = 590 nm using a UV-Vis spectrophotometer. The results revealed a strong antimicrobial effect of the thymol-containing material in the tested targets, *E. coli* and *B. subtilis*, in contrast to thyme extract, which did not provide meaningful responses compared to the control [151]. 

In addition to the critical issues linked to the functionalization processes and migration, the discrepancies in the performance of AIs alone or after the development of the materials could also be addressed by the higher concentrations used during in vitro evaluation of the neat AIs, which could overestimate their real potential. However, while the addition of AIs affects the technological properties of the PLA matrix, the constitution of active materials limits this approach, representing an important test bench for these extracts and active ingredients.

Regarding food applications, the in vitro antimicrobial properties of PLA/AI active materials were mainly investigated on *E. coli*. The presence of this bacterium in food is associated with poor hygiene during food manufacturing processes, resulting in infections associated with diarrheal symptoms [12]. The few works that included tests on fungi showed high heterogeneity in the choice of the targets, such as *B. cinerea* [117], *Candida albicans* [154], *Trichoderma* spp [150], *Saccharomyces pastorianus* [117], *Penicillum* spp., *Pestauliopsis* spp., and *Asperillus niger* [134]. The evaluations on packaged foods showed substantial differences compared to in vitro analyses. In these cases, the authors focused on general parameters such as the plate count of bacteria, both overall, mesophilic and psychrophilic, and molds and yeasts. Regarding the use of official standard methods, Llana-Ruiz-Cabello et al. referred to ISO 4833-2 [158]. This horizontal method involves the counting of aerobic microorganisms by the technique of surface insemination after incubation at 30 °C [158]. The other official method, SR ISO 6888-3:2003, mentioned only in the work of Rezaegolestani et al., is specific for coagulase-positive *Staphylococci* [161]. In general, the results showed significant antimicrobial activity of the PLA/AI packaging compared to controls, e.g., in salads [158], sausages [159,161], and catfish [110]. Altan et al. investigated the effect of electrospun nanofibers in PLA and carvacrol obtained against bacteria, molds, and yeasts present after seven days in samples of white bread. The results were proactive, with inhibitions of up to 91.3% against molds and yeasts [144].

Concerning the biomedical use of PLA/AI composite materials, one of the most promising areas is the application of wound care. Firstly, the contribution to the pollution phenomenon related to the plastic nature of disposable bandage materials; secondly, the chance, through a topical application, to emphasize the properties of natural ingredients; and finally, the biocompatibility of these materials. To strengthen the high sustainability and innovation coefficient of activated PLA in the field of wound care, electrospinning technology for the constitution of materials is used. The contamination phenomenon could be generally accounted to the proliferation of pathogens living in the surrounding microclimate. The main harmful bacterial agents are the Gram-positive *S. aureus* and the Gram-negative *E. coli* [220]. This is confirmed by the trend of antimicrobial assessments described in articles related to the application of PLA/AI materials for wound care, where those two targets were the most investigated. Regarding methods, the agar disk diffusion method was used in almost all cases. EOs could be used in the electrospinning process for the development of active PLA-based materials for wound healing uses. Zhang et al. demonstrated the inhibition effect of PLA nanofibers activated with Australian Tea Tree (*Melaleuca alternifolia* (Maiden & Betche; Cheel) EO and the anti-biofilm properties from the PLA/manuka tree (*Leptospermum scoparium*) (J.R.Forst. & G.Forst., 1776) against *Staphylococcus epidermidis* [140]. Wang et al. showed the effects of terpene-rich clary sage (*Salvia sclarea* L.) and black pepper (*Piper nigrum* L.) EOs on the development of PLA-based electrospun nanofibers. Antimicrobial test, performed according to the loss viability AATCC test method 100-2004 on *E. coli* and *S. epidermidis*, revealed promising results. PLA/black pepper EOs completely inhibited both targets, while the PLA/clary sage EO revealed a 100% and 76% antimicrobial effect on *S. epidermidis* and *E. coli*, respectively [160]. The potential uses of active PLA/AI materials in the field of medicine are not limited to antimicrobial activity. Alippilakkotte et al. described the effects of PLA/curcumin nanoparticles not only against *E. coli* and *S. aureus* but also against cancer HeLa cells with a selective cytotoxic effect when compared to the L929 control line [138]. Moreover, many natural compounds showed remarkable beneficial properties in the in vitro studies phase, but only a few of them confirmed the results in the following phases conducted in vivo [221].

### 4.5. Environmental Aspects

The biodegradation of materials derived from biomasses obtained by synthetic or biotechnological pathways is influenced by factors strictly related to the polymeric matrix, such as molecular structure, crystallization degree, and presence of AIs. In addition, environmental parameters such as temperature, humidity, pH, and the pattern of the microbial population could affect the dynamics of degradation processes [222]. 

While the biodegradation of PLA has been widely studied, that of activated materials with nanoparticles or AIs has not been thoroughly evaluated and needs further investigation [223]. 

Effectively, AIs contribute to modifying the physical-chemical properties of the polymeric matrix. However, their antimicrobial activity could even be exerted against the microbial population responsible for biodegradation and reduce the efficiency of the process. These effects could be related to the nature and concentration of the AIs, the release kinetics from the polymeric matrix, and the sensitivity of microorganisms responsible for the degradative process. 

At the same time, the investigation of PLA-based materials biodegradation in aquatic ecosystems is struggling to take off and urgently requires in-depth evaluations to prevent future pollution and track the road to the right eco-friendly development of novel polymers.

Although technological solutions from research are encouraging, the of polymers has shown that they are not emission-free and have potential impacts on greenhouse gas production and land use [224]. The main tool for assessing the environmental behavior of these materials is the life cycle assessment approach (LCA), which also considers land use change (LUC) and its global warming potential (GWP), as described in the literature [225,226,227,228]. Many results based on greenhouse gas production show that polymers can be harmful to the environment [224]. On the contrary, from an ecotoxicological point of view, recent research shows that the toxicity of these materials needs to be better researched to ensure their harmlessness to the environment. 

A preliminary study evaluating the ecotoxicological effects of PLA and PLA/starch blends on plants showed that the germination rate and plant growth of monocotyledonous and dicotyledonous plants on the resulting compost had no significant differences compared to control and remarked that PLA and PLA/starch blends are safe for the ecosystem [229]. 

A recent study evaluating the toxicity of bioplastics on polluted soils using *Allium cepa* L. showed that PLA was not phytotoxic, cytotoxic, genotoxic, or mutagenic to meristematic cells of the species tested [230]. On the contrary, a review of bioplastics and plant-based materials shows that most of them contain toxic chemicals, suggesting a more cautious approach.

In ecotoxicology, organism responses can vary significantly depending on several factors: species tested, nutritional conditions and age of organisms, chemical composition of materials tested, additives, shape and size of material, environmental conditions (e.g., temperature, pH, etc.), and interactions between materials and the complex matrix. Regarding micro and nanoplastics, many studies have been conducted recently to evaluate the factors that could affect the ecotoxicity of petroleum-based plastics in terrestrial and aquatic environments.

A recent paper shows that different species in marine ecosystems are affected differently by plastic packaging materials under natural conditions [231]. Species age and feeding conditions were of great importance in determining the effects of exposure to different types of microplastics and chemicals in freshwater (*Daphnia magna*) and in important marine species such as bacteria, algae, and echinoderms (*Vibrio fischeri*, *Phaeodactylum tricornutum*, and *Paracentrotus lividus*), as reported in the literature [232,233]. 

Recent research has also shown that other plastic exposures that were considered harmless to the environment under short-term conditions can have effects on plants exposed over a longer period and under changing environmental conditions. Furthermore, if we consider a more sensitive ecotoxicological indicator, such as biochemically induced effects, even short-term exposures can cause responses [234,235]. In addition, Fastelli and Renzi (2019) have shown that species responses to chemical exposures can vary greatly depending on environmental conditions [236], while Piccardo et al. have shown that preconditioning and interactions between plastic and the complex natural matrix can influence toxicity [237].

Some studies have shown that the environmental impact of bioplastics must also be evaluated considering the entire disposal pathway, as well as the degradation and ecotoxicity of additives and colorants contained in commercial products. Finally, it is of particular importance to perform tests under different environmental conditions, such as humidity, temperature, etc., to evaluate ecotoxicity more realistically. All the above should also be considered in the case of bioplastics to better assess whether bioplastic additives can cause ecotoxicity under different environmental conditions and to rule out their effects [238].

## 5. Conclusions and Perspectives

The journey along the state of the art of PLA-based materials described in this review confirms the chance and advantages of using natural AIs both as pure compounds and extracts from plant materials for the design and development of innovative and sustainable polymers, affecting characteristics and properties. The study has evidenced the ability of AIs to confer antimicrobial and antioxidant properties to PLA for food and biomedical applications.

Nevertheless, the outgoing scenario reveals a series of critical issues that limit the industrial scale-up. 

The use of extracts from natural sources or agro-industrial wastes represents an important key for replacing fossil-based polymers according to the green chemistry principles and circular economy strategy. However, obtaining AIs from these sources on an industrial scale requires the standardization of the extraction procedures and the chemical characterization of the extracts reported only in a few papers.

Another key factor that affects the technological transfer of lab-scale active packaging solutions is represented by the activation method. One of the most diffused processes, solvent casting, is helpful only in the starting phases of the development of new material due to its easiness and cost-effectiveness. On the other hand, the current industrial processes, such as extrusion, are mainly based on high temperatures that can compromise the chemical stability of AIs and their bioactivity. In contrast, the use of innovative techniques such as emulsification-solvent evaporation and sCO_2_ impregnation are some of the main prospects for this sector. Noteworthy, electrospinning, largely diffused in the biomedical field, represents a technique useful also in food packaging science, combining sustainability, resource optimization, and AI preservation due to the use of room temperature for processing. Moreover, electrospinning could open perspectives related to the investigation of AI combinations to obtain novel materials with enhanced antioxidant and antimicrobial properties, extending the application fields.

The reported literature suggests that further studies need to be conducted to better understand the ecotoxicological aspects, both related to the presence and release of AIs. Even though they are commonly recognized as safe, dose-dependent aspects should never be ignored to avoid adverse effects on food and humans [239]. Bioassays focused on the evaluation of the cytotoxicity of AIs and PLA-based materials could be necessary for biomedical applications [240]. Finally, LCA assessment of the whole manufacturing process represents a helpful approach to evaluating the environmental impact of novel PLA-based materials.

The multidisciplinary approach described in this review is a good model for research in this specific field, and the contribution of companies could facilitate the conversion of an academic idea into a practical solution for human health and environmental protection.
